# Suppression of CCT3 inhibits melanoma cell proliferation by downregulating CDK1 expression

**DOI:** 10.7150/jca.69497

**Published:** 2022-03-28

**Authors:** Wenlou Liu, Xiuli Zhang, Cheng Chen, Yizhi Li, Chunsheng Yang, Zhengxiang Han, Guan Jiang, Yanqun Liu

**Affiliations:** 1 Department of Oncology, Affiliated Hospital of Xuzhou Medical University, Xuzhou 221002, China.; 2 Department of Radiology, Affiliated Hospital of Xuzhou Medical University, Xuzhou 221002, China.; 3 Department of Dermatology, Affiliated Hospital of Xuzhou Medical University, Xuzhou 221002, China.; 4 Department of Dermatology, Affiliated Hospital of Xuzhou Medical University, Xuzhou 221002, China.; 5 Department of Dermatology, Affiliated Huai'an Hospital of Xuzhou Medical University, the Second People's Hospital of Huai'an, Huai'an 223002, China.

**Keywords:** melanoma, chaperonin containing TCP1, subunit 3, proliferation, apoptosis

## Abstract

The eukaryotic chaperonin family is vital for cell survival. The dysregulation of chaperonin-containing TCP-1 subunit 3 (CCT3) is implicated in several types of malignant tumors' development. However, its functional role in melanoma remains unknown. Here we elucidate the functional contribution to CCT3 to melanoma progression. The results indicated that CCT3 highly expressed in melanoma tissues, and CCT3 overexpression is correlated with clinical stage in melanoma patients. Knockdown of CCT3 by shRNA in melanoma cells inhibited cell proliferation and cell cycle progression and induced cell apoptosis *in vitro*. *In vivo*, tumor growth in the nude mice was significantly inhibited after CCT3 silencing. Importantly, the gene array analysis showed that CCT3 depletions inhibited cyclins and cell cycle regulation signaling and further evaluation demonstrated that CDK1 expression was significantly decreased after CCT3 knockdown. Additionally, Functional rescues experiments also indicated that decreased cell proliferation due to CCT3 silencing was rescued by CDK1 overexpression. Overall, our findings suggest that CCT3 depletions prohibited melanoma progression by downregulating CDK1 expression and is a potential therapeutic target for melanoma.

## Introduction

Melanoma is a highly aggressive and lethal cutaneous malignancy, and its incidence of the population is increasing steadily in recent decades 1,2. A total of 87110 new cases of malignant melanoma (MM) and 2017 witnessed 9730 deaths, accounting for 5.2% of the total cases and 1.6% of the total deaths 2. Melanoma is characterized by locore gional recurrence and distant metastasis, resulting in short survival time 3. Early stage Ⅰ melanoma has a 10‐year overall survival (OS) of 94% to 98%. However, patients with stage Ⅳ disease have a 10‐year OS of only 10% to 15% 4. Promising developments have been made in diagnosis and biomarkers, such as *BRAF, MEK* 5,6, and *KIT* 7, and treatment strategies, including immunotherapy 8,9 and molecular-targeted therapies for melanoma (such as vemurafenib, imatinib, and ipilimumab) 9-11, have rapidly advanced. However, the clinical benefits and prognosis of patients with melanoma remain dismal 12. In addition, the molecular mechanisms for melanoma development remain largely unknown. Thus, novel molecular markers must be urgently identified, and efficient therapeutic strategies must be developed to increase the survival of patients with melanoma.

Molecular chaperones play a critical role in maintaining protein homeostasis (proteostasis) and proteome integrity 13, primarily prevent the misfolding or aggregation of proteins, and help protein folding, intracellular localization, and proteolytic turnover in the cytosol 13,14. The chaperonin family includes the mitochondrial heat shock protein 60, bacterial GroEL, plastid Rubisco subunit-binding protein, and archaea group II chaperonins 15,16. The chaperonin-containing TCP-1 [TCP-1 ring complex (TRiC), CCT/TRiC] composed of eight homologous subunits (CCT1-CCT8), which is a member of the group II (eukaryotic) chaperonin family, is a large-molecular weights complex present in eukaryotic cells 17. CCT (60 kDa) features a cylindrical architecture containing two back-to-backs stacked oligomerised rings. About 10% to 15% of newly synthesized proteins were folded by CCT which can refold proteins that fail to be folded by the simple heat shock protein family 18.

CCT/TRiC is involved in various proteins concerned with oncogeneses, such as cell cycle regulatory proteins, actins, tubulins, von Hippel-Lindau tumor suppressor protein, p53, and signal transducer and activator of transcription 3 (STAT3) 19-21. The functions of CCT and its subunits such as cell proliferation, apoptosis, and tumorigenesis (including CCT1, CCT2, CCT4, and CCT8) have been assessed 22-24. However, the concrete roles of CCT3 in human cancers still remain unclear. Recently, several functions of CCT3 on tumorigenesis have been recovered by some studies. CCT3 is upregulated in HCC 16,18,25. CCT3 depletion suppresses HCC cell proliferation by inducing mitotic arrest at prometaphase and apoptosis in vitro. Clinically, the overexpression of CCT3 is associated with poor clinical prognosis and aggressive clinicopathological features 18, 25. CCT3 played a critical role in gastric cancer growth and survival 13, Peng Su et al. displayed that CCT2-CCT7 are upregulated in esophageal carcinoma 24, Shi et al. showed the significant upregulation of CCT3 in PTC samples and that CCT3 knockdown decreases cellular proliferation and cell cycle progression and induces apoptosis in K1 cellspapillary thyroid carcinoma 26, Xiong Y et al. concluded that some genes, including CCT3, COPS3 and WWP1 are candidate driver genes of importance in osteosarcoma (OS) tumorigenesis PPI network analysis 27. A prospective proteomics-based study indicated that CCT3 promises to be a biomarker for the early detection of CCA 28. Despite the adverse effect of CCT3 on the progression of different solid tumors, the involvement of CCT3 in the tumorigenesis of melanoma remains unknown.

In this study, we explored the expression of CCT3 in patients with melanoma and investigated its effects on melanoma cell proliferation, apoptosis and cell cycle progression and its potential mechanisms in vita by silencing the CCT3 gene. These findings will better our knowledge of CCT3's function of melanoma and the assessment of its value of tumor progression and targeted therapy in melanoma.

## Materials and methods

### Patients and samples

A human melanoma tissue microarray (TMA-ME803a) that contains 40 patient samples was purchased from Genechem Co.,Ltd. Shanghai, China. Within the cohort are 40 melanoma tissues, 30 adjacent normal tissues, and 10 skin tissues. No patient in the cohort received preoperative anti-tumor treatments before surgery. Written informed consent was obtained from each participant in accordance with the institutional guidelines. This study was conducted with the approval of the Ethical Review Committee of the Affiliated Hospital of Xuzhou Medical University. All methods were carried out according to the approved guidelines on Xuzhou Medical University and the researches were implemented with reference to the provisions of the Helsinki Declaration of 1975. The major clinicopathological characteristics of the patients are summarized in Table 1.

### Immunohistochemistry and evaluation

Immunohistochemistry (IHC) was conducted in accordance with a previously described method involving streptavidin peroxidase 18. In brief, the TMA slides were deparaffinized and dehydrated using a graded series of ethanol and distilled water solutions. At room temperature, 3% H_2_O_2_ was initially used to block the slides to inhibit endogenous peroxidases for 30 mins and then with 5% normal goat serum at ambient temperature for 30 mins. The slides were incubated with the primary antibody polyclonal rabbit anti-CCT3 (1:200, HPA006543; Abcam, Cambridge, MA, USA) at 4 °C overnight. The negative controls were not incubated with the primary antibody. The extent and intensity of CCT3 immunostaining were considered. The intensity of CCT3 expression was graded as follows: 0, negative; 1, weak; 2, moderate; and 4, strong. The extent of staining was grouped according to the percentage of high-staining cells in the cancer nest: 0, negative; 1, 1%-25%; 2, 26%-50%; 3, 51%-75%; and 4, 76%-100%. Multiplying the two scores was the final quantitation of each staining. The immunostaining score of a tissue section was expressed as the product of its intensity and positive percentage scores. Scoring was performed in a blinded manner and determined independently by two expert pathologists. An immunostaining score of ≤ 6 was arbitrarily grouped as low CCT3 expression, and a score of > 6 indicated high CCT3 expression.

### Cell lines and culture conditions

The human melanoma cell lines MUM-2C, MUM-2B, and A375 were acquired from the Shanghai Institute of Biochemistry and Cell Biology, Chinese Academy of Sciences (Shanghai, China). The cells lines were cultured in Dulbecco's modified Eagle's medium (RPMI-1640; Invitrogen, Thermo Fisher Scientific, Inc., Shanghai, China) provided with 10% fetal calf serum (Invitrogen), 100 U/ml penicillin, and 100 μg/ml streptomycin at 37 °C in a humidified incubator with 5% CO_2_.

### Target gene RNA interference lentiviral vector preparation

A short hairpin RNA (shRNA), shCCT3, was designed on the foundation of the target sequences 5′-CAAGTCCATGATCGAAATT-3′. A DNA oligonucleotide, which contained a target hairpin structure, transcription termination sequence, and proper restriction sites, was synthesized and inserted into the multiple cloning sites of the antiviral vector GV115 (GeneChem, Shanghai, China). Using the same technology, a control antiviral vector containing the hairpin sequence unrelated to the target was constructed. The sequence was used to confirm the antiviral shRNA constructs which were applied to lentivirus production. Using an antiviral expression system (GeneChem, Shanghai, China), virus packaging and purification were performed. Finally, 293T cells and ELISA assay (HIV-1 p24 antigen) were used to check the GFP expression of lentivirus.

### Cell transfections

Human melanoma MUM-2C and A375 cells were cultured in RPMI-1640 medium containing 10% fetal bovine serum at 37°C with 5% CO_2_. Lentivirus infection was performed on cells at 80% confluence of the condition of a multiplicity of infection (MOI) of 10. After transfection for 72 h, the number of cells expressing green fluorescent protein (GFP) was observed by fluorescence microscopy to evaluate the efficiency of lentivirus infection 13. RT-qPCR and Western blot analysis were used to detect the CCT3 gene and protein expressions in infected cells.

### RNA extraction and quantitative real time PCR assay

Total RNA in the tissue samples and melanoma cell lines were extracted and purified using the Trizol reagent (Invitrogen; Thermo Fisher Scientific, Inc.). cDNAs were synthesized by means of the Reverse Transcription Kit (Takara, Dalian, China) was synthesized. Relative quantification of RNA levels was identified by the Power SYBR Green (Takara, Dalian, China) according to manufacturer's instructions. GAPDH was chosen as an internal control for messenger RNA assays, the CT (2^-∆∆Ct^) method was utilized for calculating the relative gene expression 25. Each measurement was independently performed in triplicate. The primers used for qRT-PCR are represented as follows: CCT3 (forward) 5ʹ-TCAGTCGGTGGTCATCTTTGG-3ʹ, and (reverse) 5ʹ-CCTCCAGGTATCTTTTCCACTCT-3ʹ, GAPDH (forward) 5ʹ-TGACTTCAACAGCGACACCCA-3ʹ, and (reverse) 5ʹ-CACCCTGTTGCTGTAGCCAAA-3ʹ. The thermocycling program consisted of holding at 95 °C for 30 s, 45 cycles of 95 °C for 5 s, and 60 °C for 30 s.

### High‑content screening (HCS) for cell growth

Cell growth was evaluated with the help of the Cellomics ArrayScan HCS system (Thermo Fisher Scientific, Pittsburgh, PA, United States) 26. Briefly, A375 and MUM-2C cells were infected with lentivirus-mediated CCT3-shRNA or NC-shRNA, and the cells were seeded in 96-well plates at a density of 2 × 10^3^ cells/well, then the cells were incubated in a humidified atmosphere with 37°C and 5% CO_2_. Using the ArrayScan HCS 2.0 software (Cellomics; Thermo Fisher Scientific, Inc.), the infected GFP-expressing cells were imaged and quantified every day for five consecutive days. The experiment was performed independently more than three times. The curves depicting the infected cells growth were constructed.

### MTT assay for cell proliferation

Cell proliferation was analyzed through the MTT assay. In brief, A375 and MUM-2C melanoma cells with NC lentivirus or CCT3-shRNA lentivirus were seeded on 96-well plates (2 × 10^4^ cells/well in triplicate) and placed in a 5% CO_2_ incubator maintained at 37 °C for 1, 2, 3, 4, and 5 days. 3-(4,5-dimethylthiazolyl-2)-2,5-diphenyltetrazolium bromide (MTT) was purchased from Genview (Houston TX, USA; JT343). MTT assay was conducted following the manufacturer's instructions. The absorbance at 490 nm (OD490) was then used as a representation of cell number which was used to describe the cell growth curve. This assay was performed independently three times, and similar results were obtained.

### Apoptosis assay

According to the manufacturer's instructions, early apoptosis was tested by the annexing V-allophycocyanin (APC) apoptosis detection kit (eBioscience; Thermo Fisher Scientific, Inc.). In brief, A375 and MUM-2C cells infected with the NC or the CCT3-shRNA lentivirus were nurtured in six-well plates. After incubating for 48 h, the cells were collected, washed twice with ice-cold PBS,and cell densities were adjusted to 1 × 10^6^-1 × 10^7^/ml. Subsequently, 5 µl of annexing V-APC was added into the cell suspensions, and the cells were incubated at room temperature for 15 mins. Finally, the samples were analyzed by the FACSCalibur flow cytometer (BD Biosciences, Franklin Lakes, NJ, USA),and the data were analyzed using the FlowJo software 25. Furthermore, the case-based 3/7 activity of melanoma cells was appraised by the Caspase-Glo 3/7 assay kit (G8091, Promega, USA) as the manufacturer's instructions.

### Cell cycle assay

A375 and MUM-2C cells were infected with the NC or the CCT3-shRNA lentivirus and cultured in RPMI-1640 medium supplemented with 10% FBS in 6-well plates for two days. The cells were then trypsinized, washed with PBS, and fixed in 70% ethanol at 4°C overnight. After being washed with PBS, the cell staining solution was prepared according to the manufacturer's instructions of the cell cycle kit (Keygen Biotech Co., China), then propidium iodide (PI) staining solution was added to each tube of cell samples, resuspended the cell precipitation, and incubated at 37°C in the dark for 30 minutes. Cell cycle data were collected with FACSCalibur flow cytometer (BD Biosciences, Franklin Lakes, NJ, USA) and analyzed with ModFit LT 3.0 (Verity Software House Inc., Topsham, ME).

### Colony formation assay

The colony formation assay was conducted as described before 3. In brief, three days after lentivirus infection, 8 × 10^2^ cells suspended in DMEM containing 10% fetal bovine serum were seeded in six-well plates. The plates were incubated at 37 °C in a 5% CO_2_ incubator for 14 days or until colonies with more than 50 cells can be counted. Each dose was performed in triplicate, and the experiment was conducted at least three times.

### Western blot assay

The total protein was extracted from cultured melanoma cells using the RIPA buffer (Beyotime, Shanghai, China) to detect the expression level of CCT3. The protein concentration of each sample was determined using the BCA protein assay kit (Blue Skies, Shanghai, China). 10% SDS-PAGE gel electrophoresis was used for separating total proteins, then the proteins were transferred onto polyvinylidene difluoride membranes. After the membranes were blocked with 5% bovine serum albumin (BSA), the membranes were incubated with primary antibodies: mouse anti-GAPDH (1:2000, Santa Cruz, sc-2005), mouse anti-β-actin (1:10000, Abcam,ab6276), rabbit anti-CDK1 (1:300, Abcam, ab32094), rabbit anti-FOXM1 (1:400, Abcam, ab180710), rabbit anti-MCM-2 (1:1000, Abcam, ab109271), rabbit anti-NFKBIA (1:300, Abcam, ab7217), rabbit anti-PIM1 (1:100, Abcam, ab75776), and rabbit anti-SKP2 (1:300, Abcam, ab183039), rabbit anti-Bcl-2 (1:500, Abcam, ab692), rabbit anti-Bax (1:500, Abcam, ab32503), and rabbit anti-Cleaved PARP (1:1000, Abcam, ab32064). The membranes were then washed with TBST 3 times and incubated with the secondary antibody (Yeasen, Shanghai, China) for 2 h at room temperature. Bands were visualized using a chemiluminescent HRP substrate (Millipore, MA, USA) and an electrogenerated chemiluminescence imaging system (Tanon, Shanghai, China) 26.

### Gene expression profiling and data analysis

Total RNA transcriptional cDNA libraries of NC and KD groups were used to analyze global gene expression based on PrimeView Human Gene Expression Array. The raw microarray data onto PrimeView Human Gene Expression Array was analyzed by means of the GenespringGX predictor algorithm (Santa Clara, CA, USA). The genes were considered as a differential expression when their FDRs were less than 0.05 and the fold change was larger than 1.5 29. The microarray data were deposited in the Gene Expression Omnibus (GEO) database (Accession number: **GSE144788**). Analysis was performed for differentially expressed genes (*P* < 0.01 and fold change >2) by Ingenuity Pathway Analysis (IPA, Qiagen, www.ingenuity.com).

### Animal models

The animal experiment of the research was approved by the Ethical Committee of Xuzhou Medical University and followed the guidelines on animal care and protection. 6-week old male BALB/c nude mice were purchased from Shanghai Jiesijie Experimental Animals Co., Ltd. (Shanghai, China) (3). The male BALB/c nude mice were subcutaneously injected with A375 cells transfected with shCCT3 or shCtrl. Tumor volume (length × height × width) was measured with calipers at 10^th^, 15^th^, 20^th^, 25^th^, and 30^th^ days after inoculation. 25 days after the tumor cells were injected, mice were sacrificed by intraperitoneal injection of sodium pentobarbital at a concentration of 150mg/kg and the weight of the transplanted tumor was measured. The xenograft tissues were resected, fixed, and embedded in paraffin for IHC analyses. The animal experiment was carried out according to the ARRIVE guidelines.

### Statistical analysis

Quantitative values were presented as mean ± standard deviation/standard error of at least three independent experiments. The difference between the two groups was analyzed by Student's t-test, and one‑way analysis of variance was performed to analyze the difference among three or more groups. Differences between groups were compared by the Student-Newman-Keuls post hoc test. The correlations between CCT3 expression and various clinicopathological were analyzed by the Mann-Whitney U test. All statistical analyses were performed using the SPSS version 20.0 (SPSS, Inc., Chicago, IL, USA). *P* < 0.05 was used to represent a statistically significant difference.

## Results

### CCT3 is highly expressed in melanoma tissues and cells

**Figure 1:**
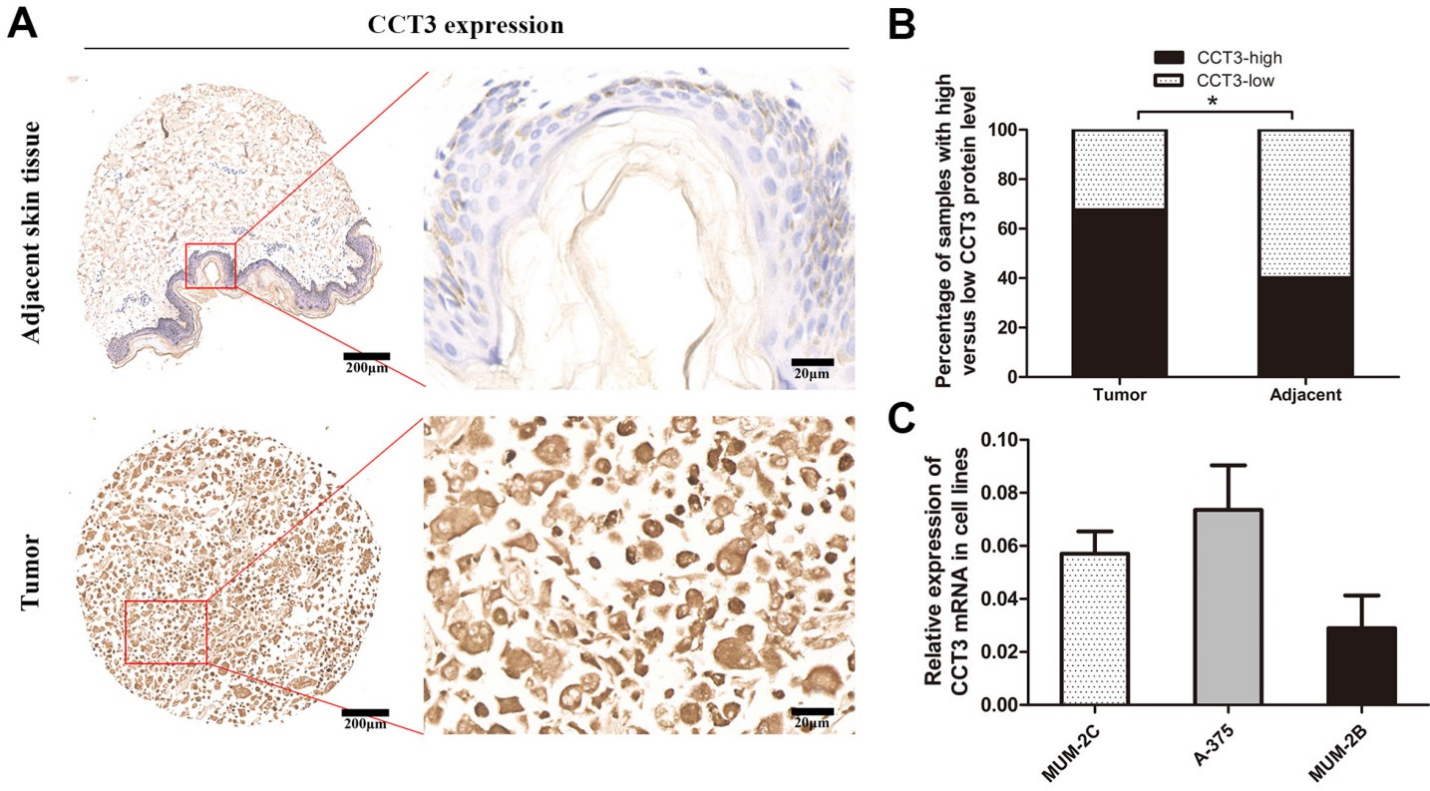
** CCT3 expression in melanoma TMAs and cell lines**. (A) Representative immunohistochemistry images of CCT3 protein expression in melanoma and adjacent non-neoplastic tissues at 40× and 400× magnifications (Scale bar: 20 *μm*)*.* Higher expression of CCT3 was observed in the cytoplasm and certain nuclei of melanoma cells compared with that of the adjacent counterparts. (B) Stacked bars indicating the percentages of melanoma samples with high and low CCT3 expression levels relative to the total number of tissues (χ^2^ test, ^*^P < 0.05). (C) Three melanoma cell lines with CCT3 mRNA expression as detected by qPCR. The 2^-ΔΔCt^ value indicates the relative expression of CCT3 in A375, MUM-2B and MUM-2C cells.

A tissue microarray of 40 melanoma patients with paired adjacent counterparts was evaluated for the expression of CCT3 protein by IHC. CCT3-positive staining was predominantly located in the cytoplasm and certain nuclei. The representative images of CCT3 expression of melanoma and adjacent normal skin tissues are shown in Figure 1A. As illustrated in Figure 1B, significantly increased CCT3 expression (CCT3-high) was found in 67.5% (27/40) of melanoma samples, whereas high CCT3 expression was only observed in 40.0% (16/40) of the paired adjacent normal skin tissues. CCT3 expression was higher in tumor tissues than that in adjacent counterparts (*P* < 0.05). Additionally, a high level of CCT3 expression was correlated with a clinical stage (*P* = 0.029, Table 1). These results indicated that CCT3 is of great importance in melanoma development. Furthermore, the expression level of CCT3 was detected in three cutaneous melanoma cell lines. qRT-PCR analysis showed that the three cell lines expressed CCT3 mRNA, and A375 and MUM-2C cells expressed more CCT3 mRNA than that of MUM-2B cells (Figure 1C). Hence, A375 and MUM-2C cells were employed for knockdown experiments.

### Downregulation of CCT3 expression by lentivirus-mediated CCT3-shRNA in melanoma cells

**Figure 2:**
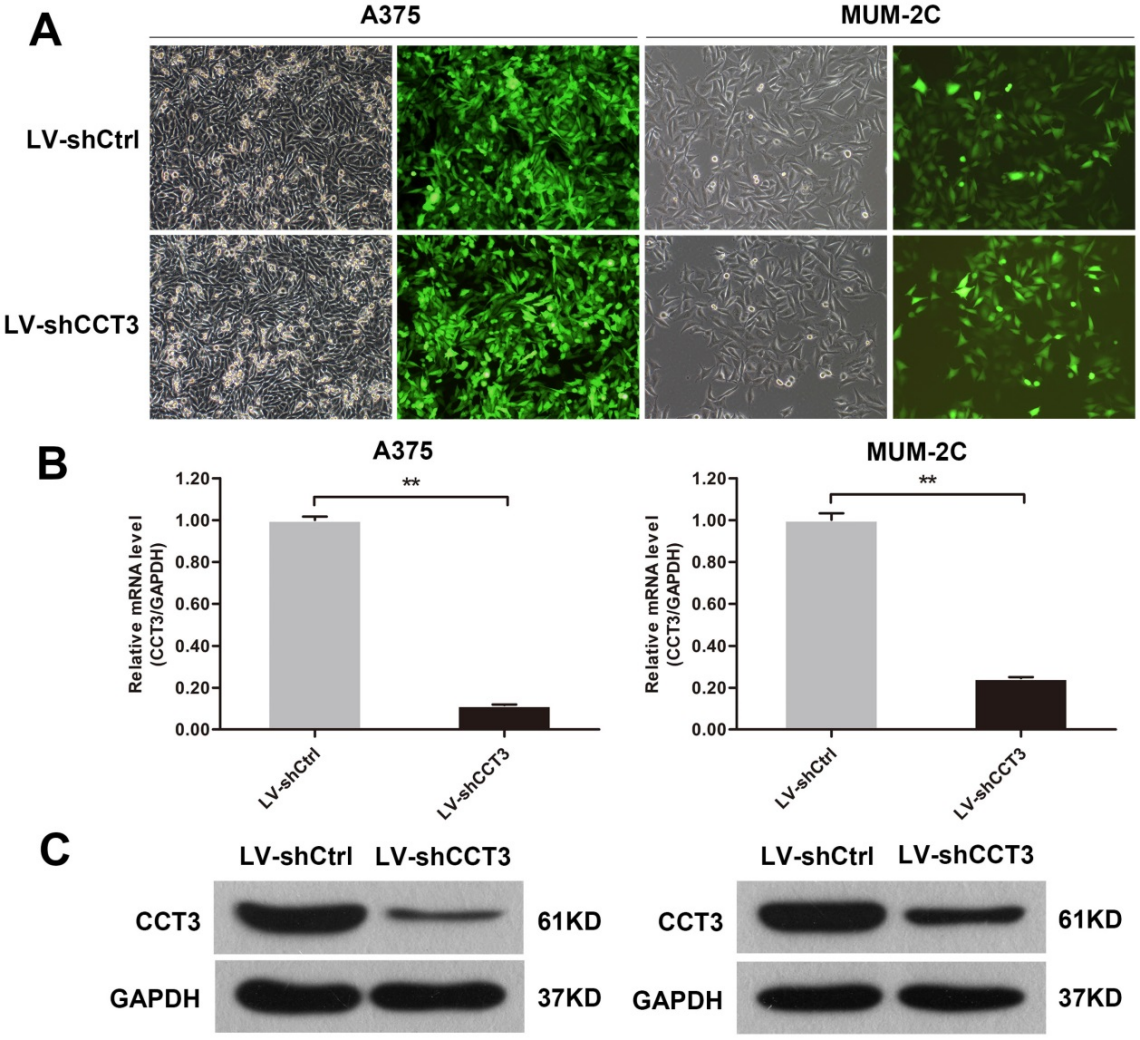
** Construction of the CCT3 knockdown melanoma cell model**. (A) Infection efficiency determined by light and fluorescence microscopy at 72 h following lentiviral infection in A375 and MUM-2C cells. Representative images of the cultures are shown (original magnification, 200×). (B) CCT3 mRNA levels in A375 and MUM-2C cells after shCCT3 lentivirus infection measured by qRT-PCR. Data are presented as mean ± SD (*n* = 3). ^**^P < 0.01, shCtrl vs. shCCT3. (C) CCT3 protein expression in the shCCT3 lentivirus-infected A375 and MUM-2C cells analyzed by Western blotting. GAPDH was used as internal control.

CCT3 was highly expressed in melanoma tissues, thus, it was inferred to promote growth and inhibit the apoptosis of melanoma cells. Cellular functions of CCT3 in melanoma cells were explored by CCT3 knockdown to verify our hypothesis. The lentivirus-expressing shRNA targeting CCT3 (LV-shCCT3) or a scramble controls shRNA (LV-shCtrl) was used to infect A375 and MUM-2C cell lines. The transfection efficiency of A375 and MUM-2C cells was verified to be above 80% by fluorometric analysis at 72 h after infection (Figure 2A). Subsequently, the efficiency of CCT3 knockdown in the two cell lines was detected by qRT-PCR. After infection, CCT3 expression decreased by 88.4% and 75.4% in A375 and MUM-2C cells, respectively, compared with that in the shCtrl group (Figure 2B). Consistently, Western blotting proved that CCT3 protein expression in A375 and MUM-2C cells was markedly lower than that in the shCtrl group (Figure 2C). These data suggested that CCT3 knockdown cell models were successfully constructed.

### Knockdown of CCT3 suppresses the proliferation of melanoma cells in vitro

**Figure 3:**
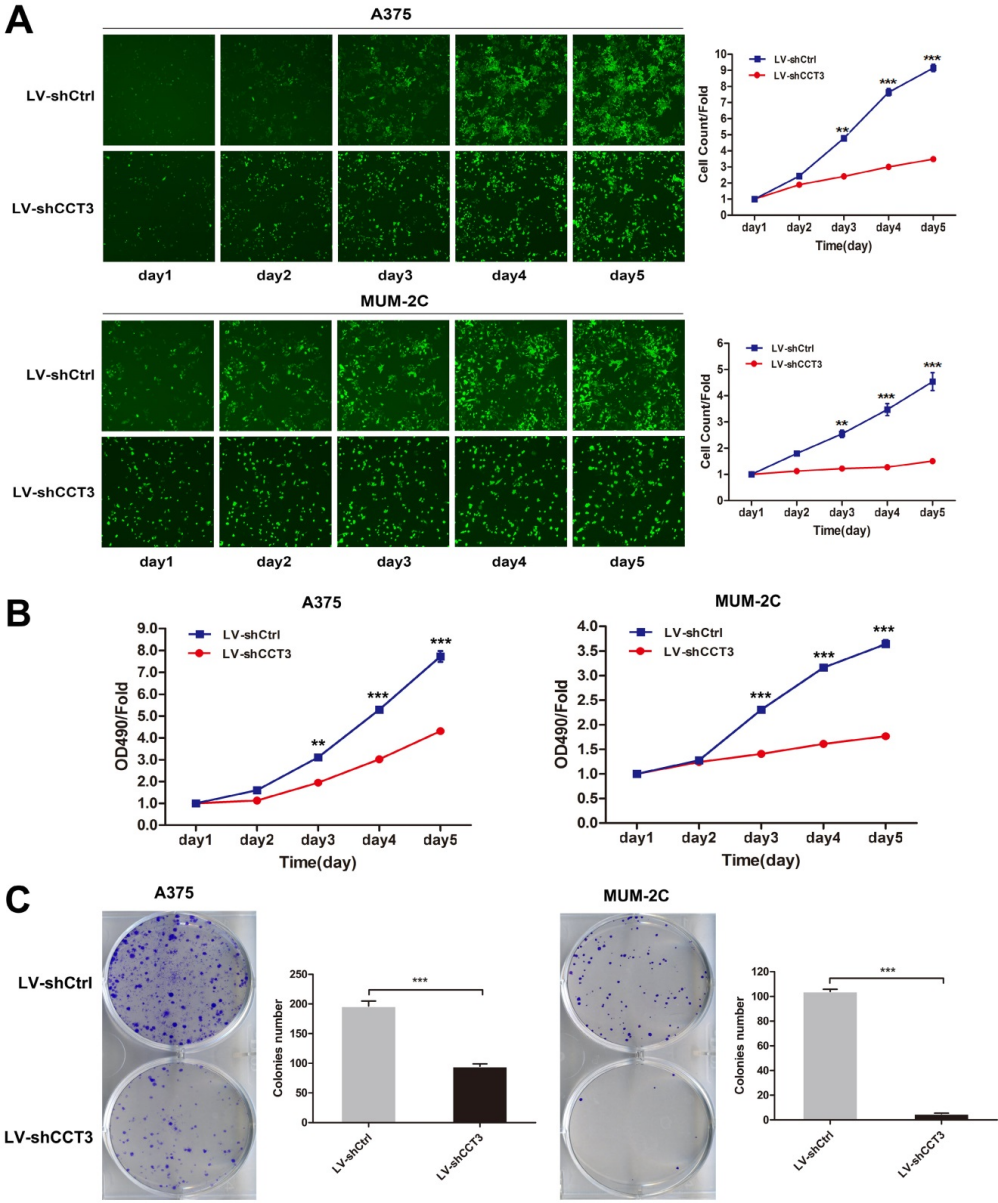
** Effect of CCT3 knockdown on the proliferation of melanoma cells**. (A) The infected cells expressing GFP imaged and counted using the Cellomics ArrayScan High Content Screening (HCS) Reader once a day for five days. The cell growth curves in shCtrl- and shCCT3-infected A375 and MUM-2C cells are shown. (B) The cell proliferation of A375 and MUM-2C cells with and without CCT3 knockdown evaluated by MTT assay. (C) Colony formation evaluated in A375 and MUM-2C cells with and without CCT3 knockdown. Data are expressed as mean ± SD (*n*=3), shCtrl vs. shCCT3, ^*^P < 0.05, ^**^ P < 0.01, ^***^ P < 0.001.

The shCtrl and shCCT3 lentivirus-infected A375 and MUM-2C cells were seeded in 96-well plates at an equal concentration to further investigate the biological effect of CCT3 knockdown on cell growth. The number of GFP-expressing infected cells was measured once a day for five days by using the Cellomics ArrayScan HCS system. As shown in Figure 3A, cell proliferation was inhibited by CCT3 silencing in a time-dependent manner. After five days of infection, the number of CCT3-shRNA-infected A375 or MUM-2C cells was significantly reduced compared with that of scr-shRNA-infected cells (*P* < 0.001). The MTT assay was conducted to further confirm the suppressive effect of CCT3 knockdown on cell proliferation. Results indicated that CCT3 knockdown in A375 and MUM-2C cells had a slower cell growth speed compared with control cells (*P* < 0.001, Figure 3B). The colony formation assay also obtained the same results. The depletion of CCT3 significantly decreased the clone formation capability of A375 and MUM-2C cells (*P* < 0.01, Figure 3C). In general, these findings indicated that CCT3 knockdown dramatically inhibits melanoma cell proliferation in vitro.

### Knockdown of CCT3 promoted melanoma cell apoptosis and influenced cell cycle progression

**Figure 4:**
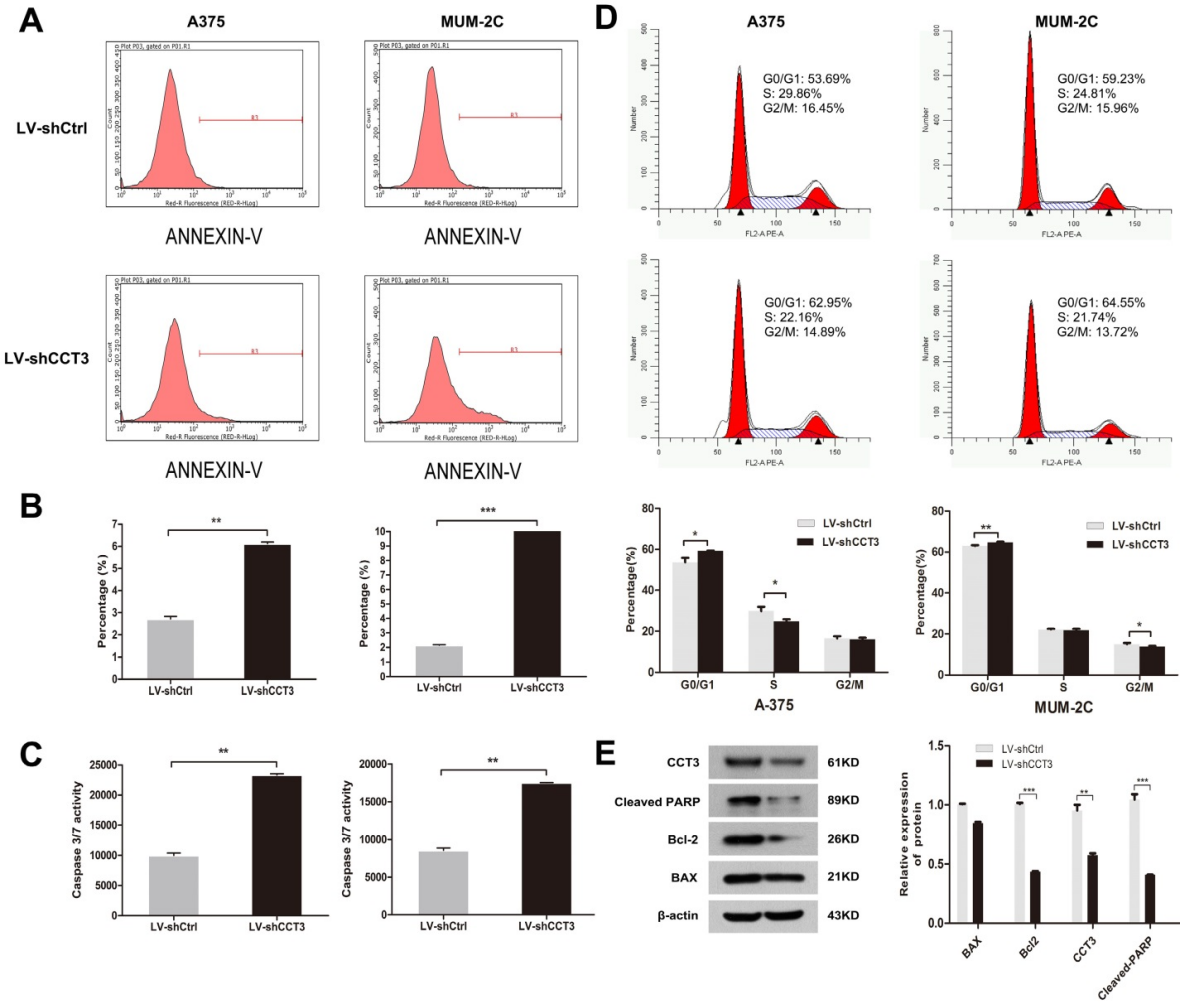
** CCT3 knockdown induces the cell apoptosis of melanoma cells and influences the distribution of cell cycle**. (A) Flow cytometry analysis based on the annexin V-APC staining for the detection of the percentage of early apoptotic cells in A375 and MUM-2C cells. Region R3 spans the fluorescence density of apoptotic cells. Representative images for each group are shown. (B) Quantification of early apoptotic cells in A375 and MUM-2C cells. (C) Activities of caspase 3/7 of A375 and MUM-2C cells with and without CCT3 knockdown detected using the caspase 3/7 activity assay. (D) Flow cytometry analysis of cell cycle distribution in A375 and MUM-2C cell lines. Representative images for each group are shown. (E) The expression levels of some downstream apoptosis markers in A375 cells by western blot. Band density was normalized to β-actin. Data are expressed as mean ± SD (*n*=3), shCtrl vs. shCCT3, ^*^P < 0.05, ^**^ P < 0.01, ^***^ P < 0.001.

Flow cytometry with annexin V-APC binding assay was performed to determine whether CCT3 knockdown affected apoptosis. CCT3 knockdown significantly facilitated cell apoptosis in A375 and MUM-2C cells compared with that in the shCtrl groups. After 96 h of shCCT3 or shCtrl lentivirus infection, 6.1% of A375 cells and 17.7% of MUM-2C cells underwent apoptosis, whereas only 2.7% and 2.2% of shCtrl cells underwent apoptosis (*P* < 0.05, Figures 4A, B). Moreover, the enhanced apoptosis of CCT3 knockdown cells was confirmed by the caspase 3/7 activity assay, which showed a 2.3- and 2.0-fold increase in caspase 3/7 activity in the shCCT3 A375 and MUM-2C cells, respectively, compared with the shCtrl cells (*P* < 0.05, Figure 4C). Cell cycle distribution analysis showed that CCT3 silencing resulted in cell cycle changes in G0/G1 phase, and a reduced percentage of cells in S-phase or G2/M phase (*P* < 0.05, Figure 4D). Finally, we detected the expression levels of some downstream apoptosis markers in A375 cells. The western blot data showed that both Cleaved-PARP and Bcl-2 were downregulated after CCT silencing (P < 0.01, Figure 4E). But Bax was not changed (*P* > 0.05, Figure 4E). These results were in accordance with the results of the MTT assay and the colony formation assay. In summary, our results demonstrated that CCT3 silencing greatly promoted apoptosis and interrupted the cell cycle progression of melanoma cells.

### Knockdown of CCT3 inhibited tumor growth in melanoma in vivo

**Figure 5:**
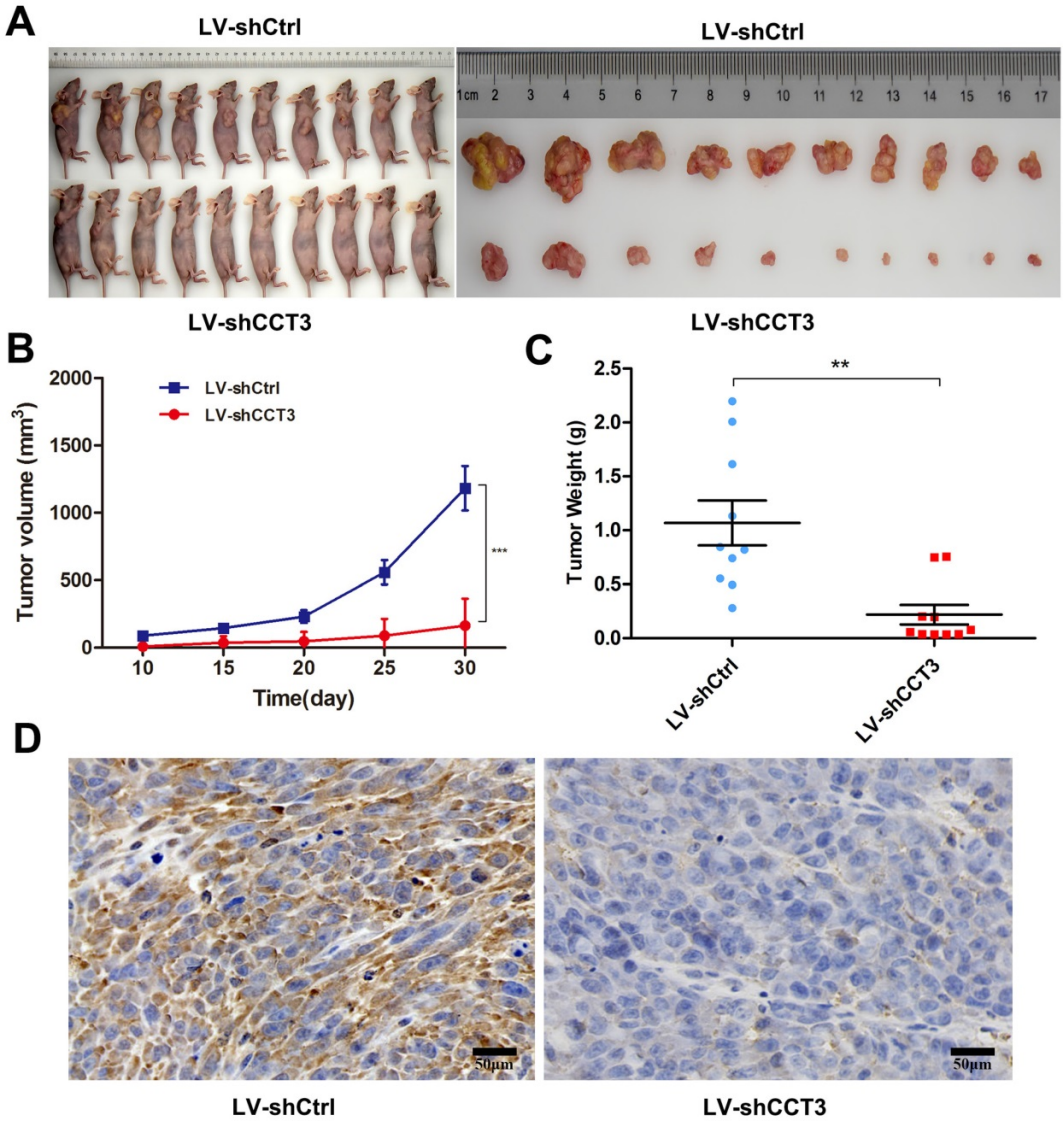
** CCT3 expression knockdown attenuated the tumorigenicity of A375 cells in vivo**. (A) Nude mice subcutaneously inoculated with A375-LV-shCCT3 and control cells to test their proliferative ability in vivo. Tumor images resected from mice in shCCT3 and shCtrl group. Average volume (B) and weight (C) of the subcutaneous xenografts in shCtrl and shCCT3 groups. (D) Immunohistochemistry results showing the expression of CCT3 in paraffin-embedded xenograft tissues (original magnification, 200×; Scale bar: 50 *μm*). Data are expressed as mean ± SEM. ^*^P < 0.05, ^**^P < 0.01, compared with shCtrl group.

The above results demonstrated that CCT3 knockdown inhibited cell proliferation, affected the distribution of cell cycles,and promoted the apoptosis of melanoma cells in vita. Melanoma xenograft mouse models were employed to investigate the effects of CCT3 silencing on tumorigenicity in vivo. A375-LV-shCCT3 and control cells were injected subcutaneously into two separate groups of BALB/c nude mice (*n* = 10). The mice were euthanized, and their tumor volume and weights were examined on the 10^th^, 15^th^, 20^th^, 25^th^, and 30^th^ days after inoculation. As shown in Figure 5A, CCT3 knockdown significantly suppressed the tumorigenicity of A375 cells. CCT3 knockdown mice xenografts had a smaller tumor volume and less weight than the control group (*P* < 0.05, Figures 5B, C). In addition, IHC analysis was conducted to detect the expression of CCT3 in xenograft tumors. Tumors treated with LV-shCCT3 exhibited significantly impaired staining of CCT3 compared with the control groups (Figure 5D), suggesting that CCT3 knockdown inhibited melanoma cell tumorigenicity in vivo.

### CCT3 is involved in the regulation of multiple signaling pathways in melanoma cells

**Figure 6:**
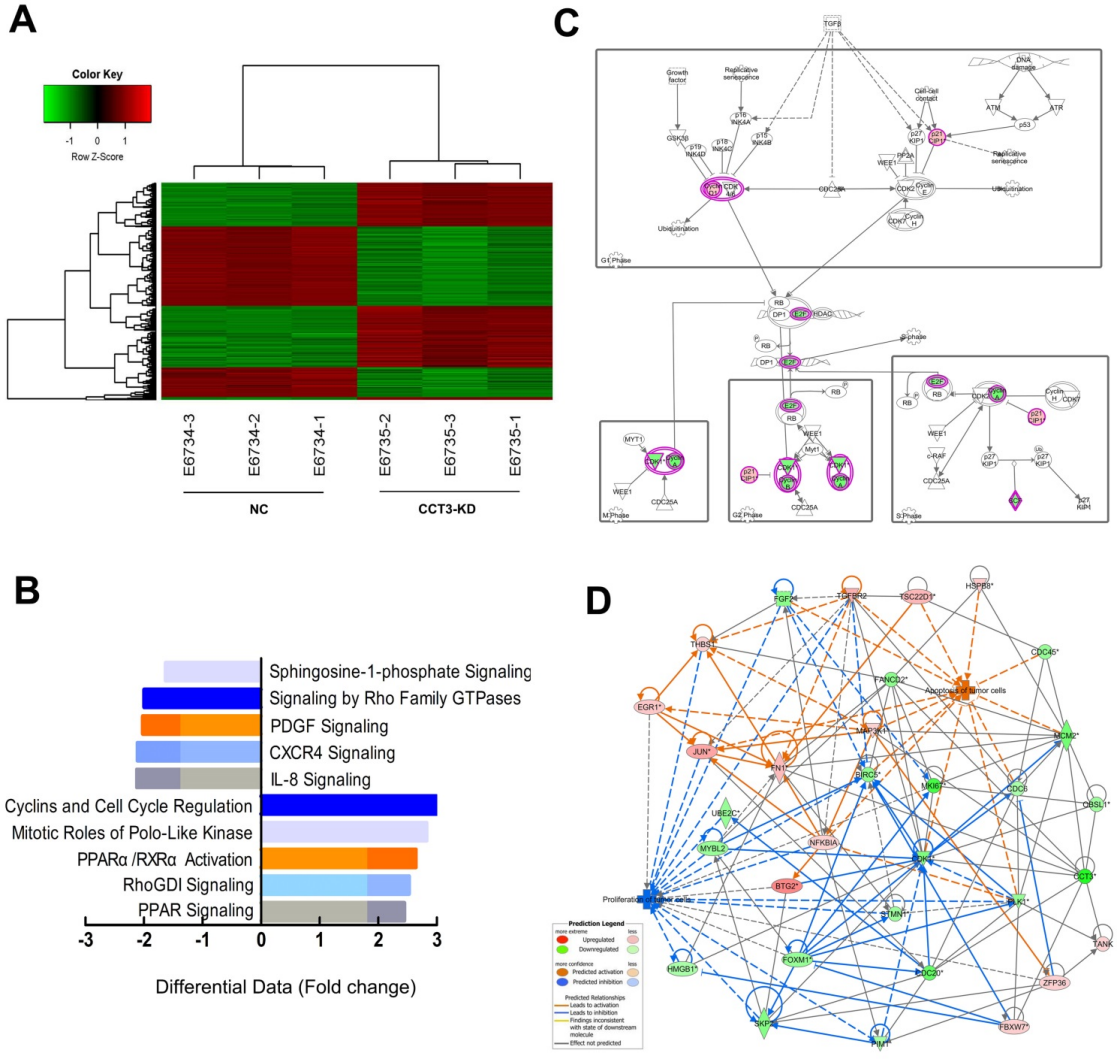
** Global changes in melanoma cells transcriptome following the knockdown (KD) of CCT3**. (A) Hierarchical cluster analysis of differentially expressed genes with fold change of > 2.0. Row and column represent gene and experimental cells, respectively. Upregulated and downregulated genes are shown in red and green, respectively. (B) Signaling enrichment analysis of CCT3-downregulated classical signaling pathways based on IPA. Differential data are presented as z-scores (based on Fold change). (C) The expression trends of molecules in cyclins and cell cycle regulation pathway (based on IPA). Red and green represent upregulated and downregulated genes, respectively. (D) Knowledge-based interaction network of CCT3 targets after comparing the CCT3-KD and the shCtrl cells. The network was built on the basis of the CCT3 interactome of microarray data with a 1.5-fold change cutoff. The intensity of the node color indicates the degree of upregulation (red) or downregulation (green). Light colors represent less significant *p* values.

We conducted the genome-wide gene expression analysis via the GeneChip PrimeView Human Gene Expression Array (Affymetrix, USA) to explore the potential molecular mechanism of CCT3 in the complicated signal pathways. According to the principles of standardization and bioinformatics analyses, two groups can clearly be distinguished through the hierarchical cluster and the principal component analyses. Considering the inclusion criteria of showing a fold change of > 2.0 and *P* < 0.05, the gene array analysis identified 497 upregulated and 500 downregulated genes in CCT3 knockdown cells (data not shown). An overview of the significant differential expressed genes (DEGs) was provided by the associated heatmap (Figure 6A). IPA (Qiagen, USA) was used to identify possible relationships and pathways relevant to the gene expression profile. Compared with shCtrl cells, cells depleted with CCT3 were related to several changes in specific signaling pathways. The pathway analysis showed that CCT3 depletion inhibited cyclins and cell cycle regulation signaling, PPARα/RXRα signaling, RhoGDI signaling, and PPAR signaling (Z-score < 0). CCT3 knockdown also activated several signaling pathways, including platelet-derived growth factor signaling pathways, IL-8 signaling, and CXCR4 signaling (Z-score>0, Figure 6B). These pathways are important in cancer cell growth, apoptosis, and survival. Among them, cyclins and cell cycle regulation signaling pathways were significantly inhibited in the CCT3-KD A375 cells compared with the control cells (Z-score > 2.5). The detailed gene alterations are shown in Figure 6C.

### Overexpression of CDK1 partially rescued the diminished proliferation abilities caused by CCT3 knockdown

**Figure 7:**
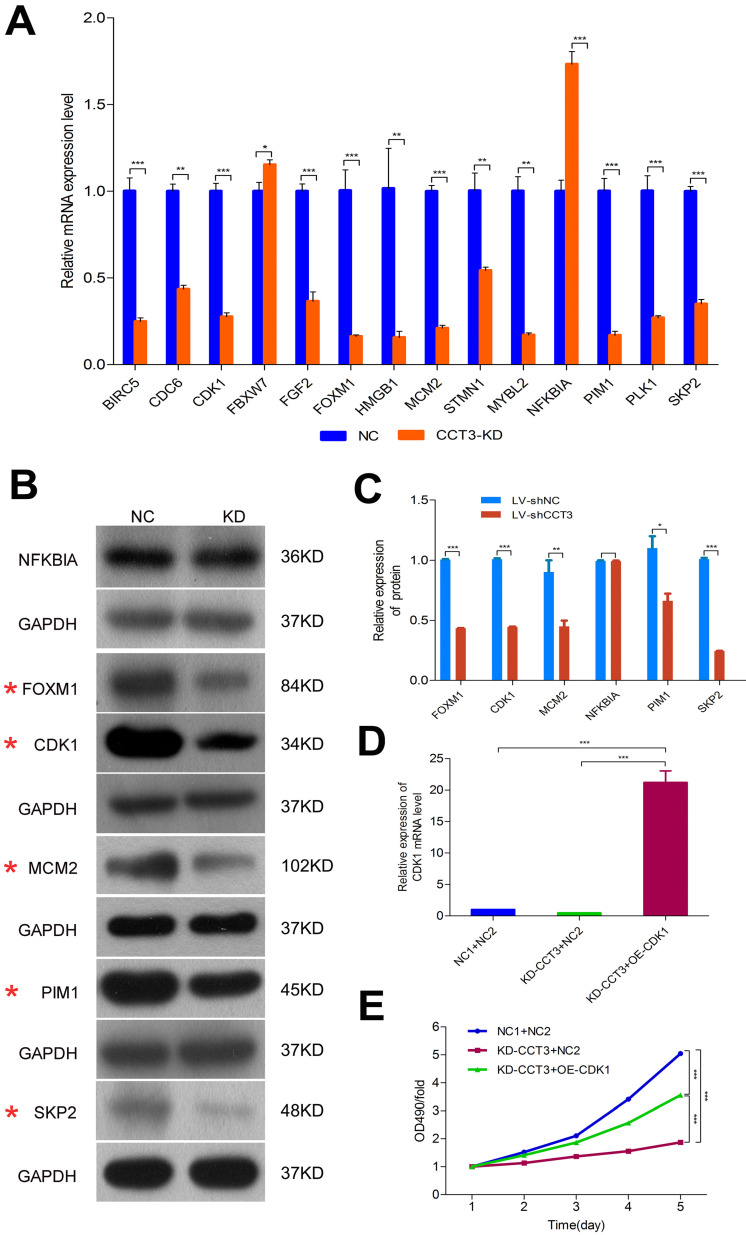
** The validation results and changes of cell cycle signal molecules after CCT3 silencing in melanoma cells**. (A) qRT-PCR showed the upregulation of 12 genes and downregulation of two genes in CCT3-KD A375 cells. (*t* test, *P* < 0.01 for all genes). (B) and (C). Western blotting analysis showed decreased levels of CDK1, FOXM1, MCM2, PIM1, and SKP2 genes and no statistical difference for NFKBIA in CCT3-KD A375 cells. (D). RT-qPCR results indicated that the expression of CDK1 mRNA was successfully rescued after shCCT3 melanoma cells were infected with CDK1 overexpression lentivirus vector (OE-CDK1). (E) MTT assay showed that decreased cell proliferation by CCT3 silencing was rescued by the overexpression of CDK1. ^*^P < 0.05, ^**^P < 0.01, ^***^P < 0.001, compared with the shCtrl cells.

An enrichment analysis of microarray data and the knowledge-based interaction network analysis were performed to identify the specific potential targets of CCT3 related to the cyclins and cell cycle regulation (Figure 6D). A total of 32 genes correlated with cyclins and cell cycle regulation were further investigated. After CCT3 depletion, the changes of gene expression were identified by qRT-PCR, and results were consistent with the microarray data except for the EGR1, MAP3K1, THBS1, and TGFBR2 genes (Figures S1-3 in Supplementary Material). Figure 7A displays the differential expression of 14 out of 32 genes, including 12 downregulated genes and 2 upregulated genes. From the 14 genes, six classical and significantly altered genes, including CDK1, FOXM1, MCM2, NFKBIA, PIM1, and SKP2 genes, were screened to further confirm the results of microarray analysis. The western blotting demonstrated that the expression levels of CDK1, FOXM1, MCM2, PIM1, and SKP2 genes in the CCT3-KD cells were significantly lower than those in the shCtrl cells (all *P* < 0.05). However, no statistical difference was found in NFKBIA (*P* > 0.05, Figure 7B, C).

CDK1, the classical DEG in the cell cycle regulation pathway, was identified for further study to determine the importance of the CDK family in regulating the cell cycle. In A375 cells, the expression level of CDK1 was significantly decreased after CCT3 knockdown, which may be suggested as a potential downstream effector of CCT3 in tumorigenesis. To confirm the causal relationship between CCT3 and its downstream targets, the functional rescue experiments were performed by treating A375 cells with shCCT3 vector alone or together with the CDK1 overexpression vector. As shown in Figure 7D, the PCR results indicated that the expression of CDK1 mRNA was successfully rescued after shCCT3 melanoma cells were infected with a CDK1 overexpression lentivirus vector. The treated cells were then subjected to proliferation assays. MTT assay also showed that decreased cell proliferation due to CCT3 silencing was rescued by CDK1 overexpression (*P* < 0.001, Figure 7E), suggesting that CDK1 may serve as a downstream effect of CCT3 on tumor cell growth in melanoma.

## Discussion

The role of molecular chaperones in the initiation and progression of cancer has been widely researched. CCT/TRiC consists of a double-ring structure, and each ring contains eight different subunits 17,30. Each subunit contributes to the complete folding of the protein and cytoskeletal components 27,30,31. CCT3, an important member of the CCT family, shares a similarity insignificant sequence with other members of the CCT family of chaperone proteins and has conserved domains similar to other distant chaperone proteins 31, 32. Previous studies have shown that many solid tumors and malignant blood diseases overexpress molecular chaperones which are regarded as a potential therapeutic target for cancer treatment 13,33,34. However, none of the former studies has discussed the tumor-inhibiting functions of CCT3 silencing in melanoma.

In this report, our study presented the first report on the tumor-promoting effect of CCT3 in melanoma cells. we demonstrated CCT3 was highly expressed in melanoma specimens compared with adjacent tissues, and the level of CCT3 is correlated with the clinical stage in melanoma patients. Knockdown of CCT3 expression in the melanoma cells suppresses cell proliferation and cell cycle progression and promote apoptosis. These findings were similar to the results of several published investigations 13,18,26,35. Subsequently, The investigation of nude mouse xenograft models strongly supported that the knockdown of CCT3 significantly suppressed tumor growth. Therefore, our results indicated that CCT3 knockdown can significantly inhibit the development of melanoma. However, the potential molecular mechanisms that regulate melanoma progression have been rarely explored.

The possible mechanism may have been elucidated that CCT3 regulates the function of HCC cells. Kasembeli et al. showed that the eukaryotic chaperonin TRiC/CCT biological activity was promoted by STAT3 in vitro and in vivo 20. Hou et al. found that the mRNA expression of CCT3 in HCC is negatively correlated with DNA methylation 31. Cui et al. showed that CCT3 knockdown may suppress the IL6/STAT3 signaling pathway 18. In the present study, exploring the potential mechanism by which CCT3 may be involved in melanoma was attempted through IPA analysis. The results indicate that the cell cycle regulation is the most important pathway related to CCT3. The occurrence and development of HCC have a close relationship with the cell cycle changes and molecular drug intervention in the cell cycle 36-38. The expression profile analysis of CCT3-related genes enriched in the cell cycle signaling pathways revealed five genes that were most relevant to CCT3: *CDK1, FOXM1, MCM2, PIM1,* and* SKP2*. CDK family is important in the tumor cell cycle and tumor proliferation. It has been reported that CCT family and CDK family are closely related. Li et al. reported that CCT3 knockdown is associated with the upregulation of CDK2 and CDK6 13. Huang et al. revealed that CCT8 knockdown dramatically reduces the level of CDK2 in HCC cells 39. CDK1 is highly expressed in HCC cells and activates the cell cycle pathway to promote tumor cell proliferation 40. In our study, the expression of CDK1 was downregulated in shCCT3 melanoma cells. Therefore, we chose classical CDK1 to study its relationship with CCT3. Both qRT-PCR and Western blotting were indicated that CCT3 knockdown significantly decreased CDK1 expression. The reciprocal experiment also showed that the decreased cell proliferation by CCT3 knockdown was rescued by the overexpression of CDK1. Then, these results may imply that CCT3 silencing regulates the expression of CDK1, and affects the process of the cell cycle, resulting in the inhibition of proliferation of melanoma cells. CDK1 can serve as a potential downstream target of CCT3 in melanoma. However, only CDK1 was selected for this research, whether the other four genes in WB verification were functionally regulated by CCT3 in melanoma cells needs further and in-depth study.

In conclusion, our data demonstrated that CCT3 plays an important role in the development and progression of melanoma. The tumor-inhibiting functions of CCT3 silencing in melanoma were associated with CDK1 downregulation in the cell cycle signaling pathway, which might provide novel markers and targets for melanoma diagnosis and treatment.

## Supplementary Material

Supplementary figures.

## Figures and Tables

**Table 1:** Correlation between CCT3 expression and clinicopathological characteristics of patients with melanoma

Characteristics	Number of Patients		P value
	Low CCT3 expression (n)	High CCT3 expression (n)	
Age (years)			
< 60	7	11	0.435
≥ 60	6	16	
Sex			
Male	5	9	0.750
Female	8	18	
Invasion			
T0-T2	8	13	0.427
T3-T4	5	14	
Lymph nodes metastasis			
No	7	10	0.314
Yes	6	17	
Clinical stage			
I-II	8	7	0.029
III-IV	5	20	
Distant metastasis			
No	9	12	0.142
Yes	4	15	

Note: A chi-square test was used for comparing groups between low and highCCT3 expression. * p < 0.05 was considered significant.

## Abbreviations

CCT3: Chaperonin-containing TCP-1 subunit 3
CDK1: Cyclin-dependent kinase 1
GEO: Gene Expression Omnibus
GFP: Green fluorescent protein
HCS: High‑content screening
HCC: Hepatocellular carcinoma
IHC: Immunohistochemistry
RT-qPCR: Quantitative real time PCR
STAT3: Signal transducer and activator of transcription 3
TMA: Tissue microarray
