# Supplementary material for: Suppression of CCT3 inhibits melanoma cell proliferation by downregulating CDK1 expression
Source: J Cancer. 2022 Mar 28;13(6):1958–71. doi: 10.7150/jca.69497 (PMC8990421; doi:10.7150/jca.69497)
Supplement: Supplementary file 1 — Supplementary figures. [file jcav13p1958s1.pdf]

Supplementary materials: A total of 32 genes correlated with cyclins and cell cycle regulation were further investigated (Figures S1-3).

Figures S1

A

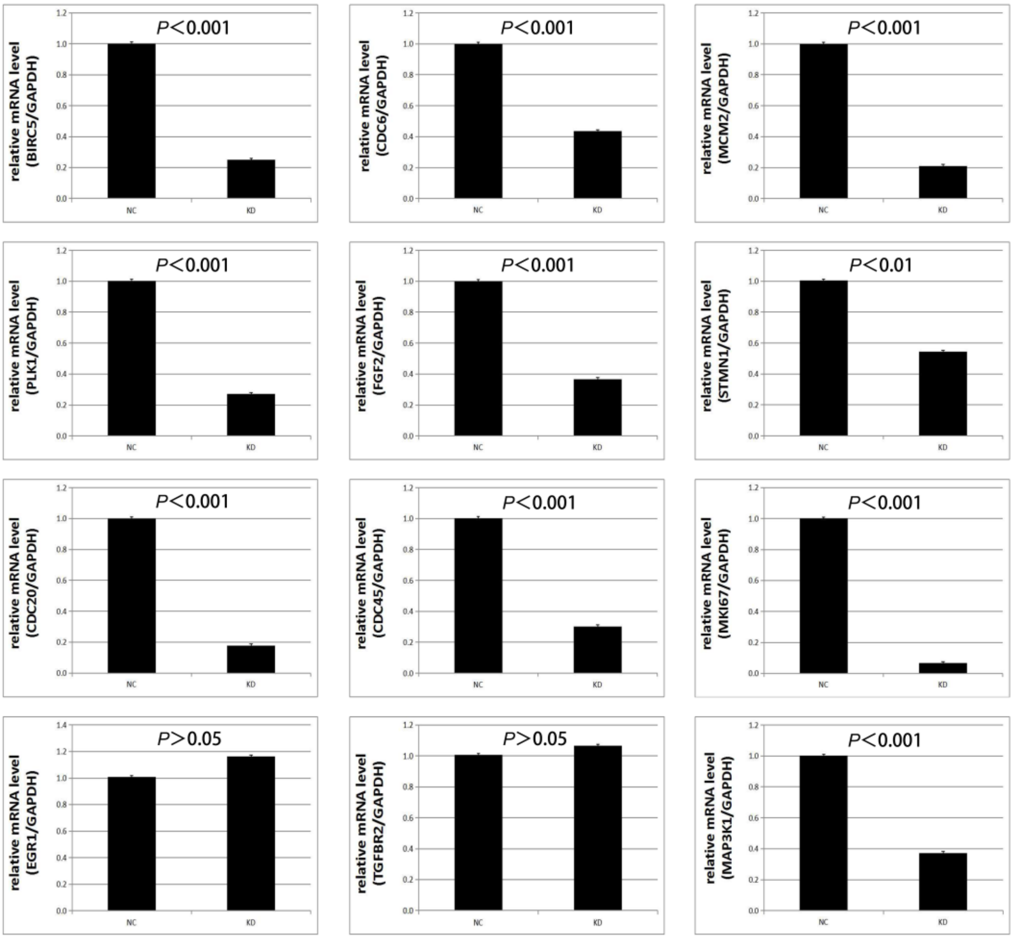

Figures S2

B

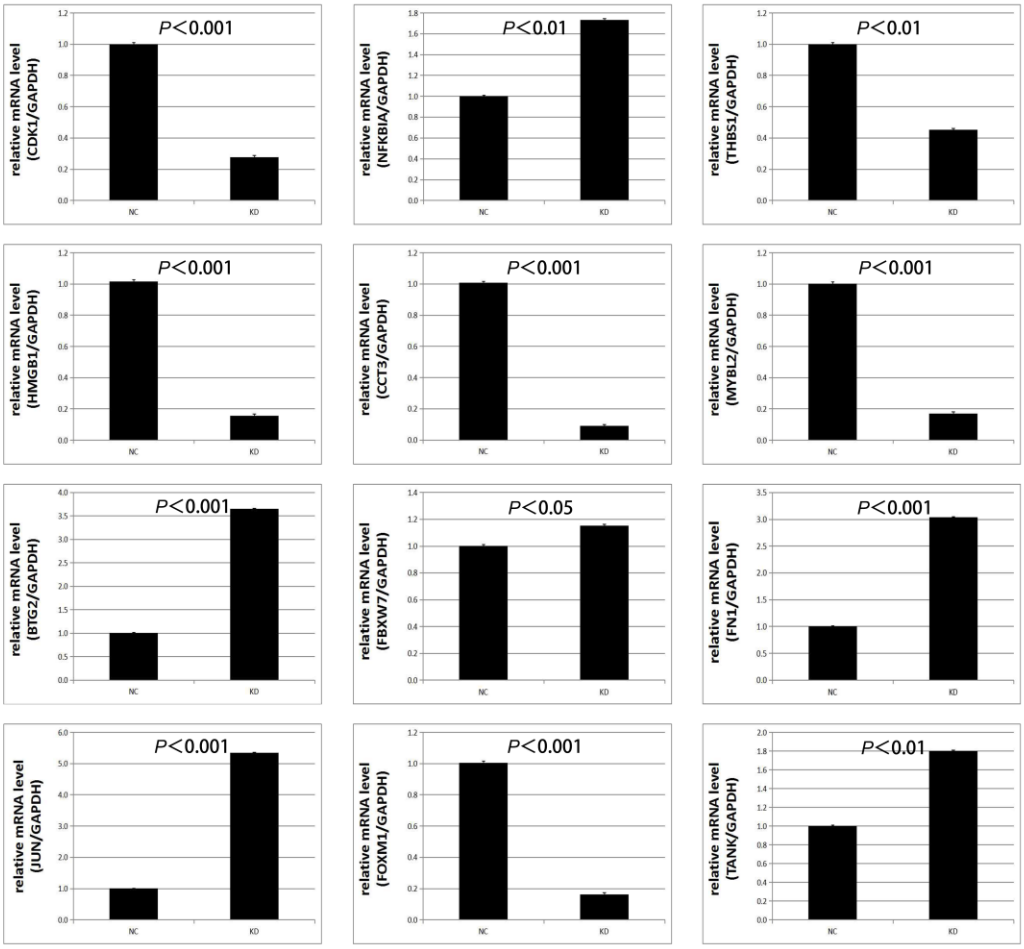
P<0.001
relative mRNA level (CDK1/GAPDH)
P<0.01
relative mRNA level (NFKBIA/GAPDH)
P<0.01
relative mRNA level (THBS1/GAPDH)
P<0.001
relative mRNA level (HMGB1/GAPDH)
P<0.001
relative mRNA level (CCT3/GAPDH)
P<0.001
relative mRNA level (MYBL2/GAPDH)
P<0.001
relative mRNA level (BTG2/GAPDH)
P<0.05
relative mRNA level (FBXW7/GAPDH)
P<0.001
relative mRNA level (FN1/GAPDH)
P<0.001
relative mRNA level (JUN/GAPDH)
P<0.001
relative mRNA level (FOXM1/GAPDH)
P<0.01
relative mRNA level (TANK/GAPDH)
NC
KD

Figures S3

C

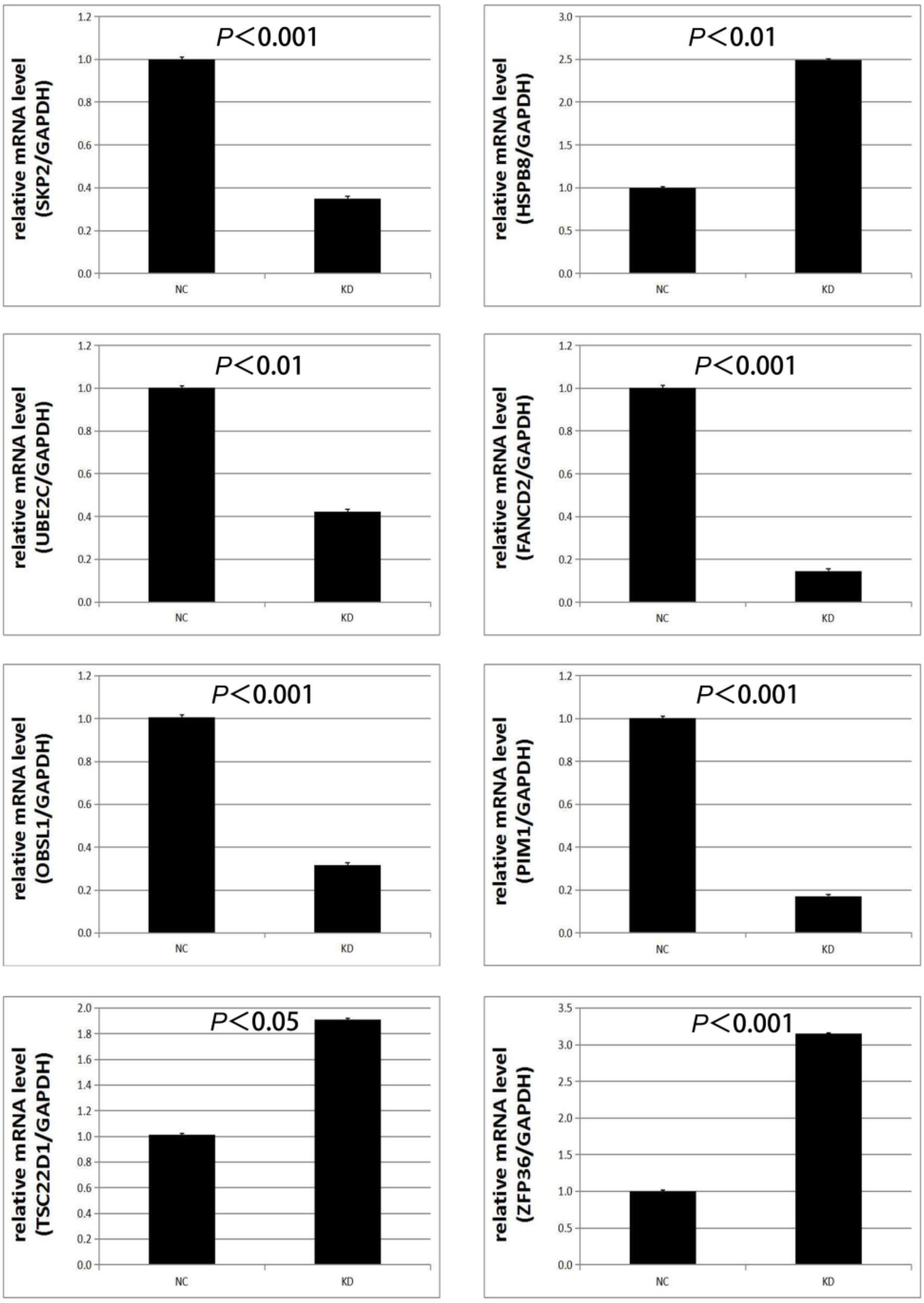
relative mRNA level (SKP2/GAPDH)
$P < 0.001$
relative mRNA level (HSPB8/GAPDH)
$P < 0.01$
relative mRNA level (UBE2C/GAPDH)
$P < 0.01$
relative mRNA level (FANCD2/GAPDH)
$P < 0.001$
relative mRNA level (OBSL1/GAPDH)
$P < 0.001$
relative mRNA level (PIM1/GAPDH)
$P < 0.001$
relative mRNA level (TSC22D1/GAPDH)
$P < 0.05$
relative mRNA level (ZFP36/GAPDH)
$P < 0.001$
NC
KD
